# Early Prognostic Value of the Freiburg Index of Post-Transjugular Intrahepatic Portosystemic Shunt Survival in Patients With Hepatocellular Carcinoma Undergoing Liver Transplantation

**DOI:** 10.7759/cureus.116078

**Published:** 2026-09-10

**Authors:** Yifan Ling, Jiaxin Zhao, Mengni Jing, Yujiang Pang, Jie Zhou, Zhenhua Hu

**Affiliations:** 1 Department of Surgery, The Fourth Affiliated Hospital, School of Medicine, Zhejiang University, Yiwu, CHN; 2 Department of Surgery, The First Affiliated Hospital, School of Medicine, Zhejiang University, Hangzhou, CHN; 3 Division of Hepatobiliary and Pancreatic Surgery, Department of Surgery, The First Affiliated Hospital, School of Medicine, Zhejiang University, Hangzhou, CHN; 4 Department of Surgical Oncology, The First Affiliated Hospital, School of Medicine, Zhejiang University, Hangzhou, CHN

**Keywords:** freiburg index of post-tips survival, hepatocellular carcinoma, liver transplantation, overall survival, recurrence

## Abstract

Background: The Freiburg Index of Post-Transjugular Intrahepatic Portosystemic Shunt (TIPS) Survival (FIPS) integrates clinically relevant indicators of hepatic and renal function, but its prognostic value in patients with hepatocellular carcinoma (HCC) undergoing liver transplantation (LT) remains unclear. This study aimed to evaluate the association of pre-transplant FIPS with post-transplant overall survival (OS) and HCC recurrence and to compare its prognostic performance with that of the Model for End-Stage Liver Disease (MELD).

Methods: Adult liver transplant recipients with HCC registered in the Scientific Registry of Transplant Recipients (SRTR) between January 1, 2010, and December 31, 2018, were included. Overall survival was evaluated using Kaplan-Meier analysis and multivariable Cox regression. Because FIPS did not satisfy the proportional hazards assumption, interval-specific effects were estimated for 0-1, 1-2, and 2-3 years after transplantation. Parallel FIPS- and MELD-based prognostic models were compared using Harrell's C-index, time-dependent area under the curve (AUC), calibration analysis, decision-curve analysis, and five-fold cross-validation. HCC recurrence was evaluated using Fine-Gray competing-risk regression, with death without recurrence treated as a competing event.

Results: Patients with high FIPS had consistently lower post-transplant survival than those with low FIPS, with one-, two-, and three-year overall survival rates of 89.5%, 84.7%, and 79.6% versus 93.1%, 88.3%, and 83.8%, respectively. After multivariable adjustment, each one-unit increase in FIPS was significantly associated with higher mortality during the first year after transplantation (hazard ratio (HR): 1.125; 95% confidence interval (CI): 1.069-1.183; P<0.001) and during years 2-3 (HR: 1.112; 95% CI: 1.040-1.190; P=0.002), demonstrating a time-dependent prognostic association. A sensitivity analysis excluding Milan criteria included 13,940 recipients and yielded directionally consistent associations, with significant estimates across all three follow-up intervals. FIPS-based risk prediction showed performance comparable to that of MELD across cross-validated C-index, time-dependent AUC, calibration, and decision-curve analyses. In competing-risk analysis, high FIPS showed a trend toward a higher risk of HCC recurrence (subdistribution hazard ratio (sHR): 1.22; 95% CI: 0.97-1.54; P=0.095).

Conclusion: Pre-transplant FIPS captures time-dependent variation in early post-transplant survival among patients with HCC and provides prognostic information comparable to MELD. These findings support the potential use of FIPS as a complementary marker for pre-transplant risk stratification and early post-transplant prognostic assessment. Its relationship with post-transplant HCC recurrence warrants further investigation.

## Introduction

Hepatocellular carcinoma (HCC) is the most common primary liver malignancy and remains a major cause of cancer-related mortality worldwide [1,2]. It commonly arises in the setting of chronic liver disease and cirrhosis, particularly those related to viral hepatitis, alcohol-associated liver disease, and metabolic liver disease [2]. Curative-intent treatment options include hepatic resection, local ablation, and liver transplantation (LT), whereas locoregional and systemic therapies are used according to tumor stage, liver function, and transplant eligibility [3,4]. Among appropriately selected patients, LT is particularly valuable because it treats both the tumor and the underlying diseased liver.

Despite advances in candidate selection and perioperative management, post-transplant outcomes remain heterogeneous, and accurate pre-transplant risk stratification is important in the setting of limited donor-organ availability [5,6]. The Model for End-Stage Liver Disease (MELD) is widely used to quantify liver disease severity and guide liver-allocation priority, but it was not specifically developed to characterize post-transplant prognosis in recipients with HCC [7,8]. Accordingly, additional pre-transplant markers that capture hepatic, renal, and systemic reserve may provide complementary prognostic information in this population.

The Freiburg Index of Post-Transjugular Intrahepatic Portosystemic Shunt (TIPS) Survival (FIPS), originally developed by Bettinger et al. to predict survival after TIPS, incorporates age, serum bilirubin, creatinine, and albumin [9]. Subsequent studies have validated its prognostic utility in patients with cirrhosis undergoing TIPS and have shown performance comparable or superior to several conventional liver-disease scores in that setting [10,11]. Because the components of FIPS reflect hepatic function, renal function, nutritional status, and age, the score may also capture clinically relevant physiologic reserve before LT. However, its prognostic value in patients undergoing LT for HCC has not been well defined.

Using a large Scientific Registry of Transplant Recipients (SRTR) cohort, this study evaluated the association of pre-transplant FIPS with early overall survival (OS) and post-transplant HCC recurrence. We further characterized the time-dependent effect of FIPS and directly compared the prognostic performance of parallel FIPS- and MELD-based models. The study was designed to assess whether FIPS could provide useful complementary information for pre-transplant risk stratification in patients undergoing LT for HCC.

## Materials and methods

This retrospective cohort study used data from the Scientific Registry of Transplant Recipients (SRTR), which includes information submitted by members of the Organ Procurement and Transplantation Network (OPTN) in the United States. OPTN and SRTR activities are overseen by the Health Resources and Services Administration (HRSA), US Department of Health and Human Services. The data reported in this study were supplied by the Hennepin Healthcare Research Institute as the SRTR contractor. The interpretation and reporting of these data are the responsibility of the authors and do not represent official policies or interpretations of SRTR or the US government. The study protocol was reviewed and approved by the Ethics Committee of Zhejiang University, China.

Adult patients who underwent liver transplantation for HCC between January 1, 2010, and December 31, 2018, were retrospectively identified. Eligible recipients were aged ≥18 years and had a primary listing diagnosis of hepatocellular carcinoma or liver cancer. Patients were excluded if they were younger than 18 years, had undergone prior liver transplantation, had a primary diagnosis of benign liver disease or a liver tumor other than HCC, or lacked data required to calculate FIPS. Among 15,924 recipients transplanted for HCC, 186 were excluded because one or more FIPS components were unavailable, leaving 15,738 recipients with calculable FIPS. A further 1,495 recipients were excluded because survival or death status information was unavailable. The final cohort therefore comprised 14,243 recipients (Figure 1).

**Figure 1:**
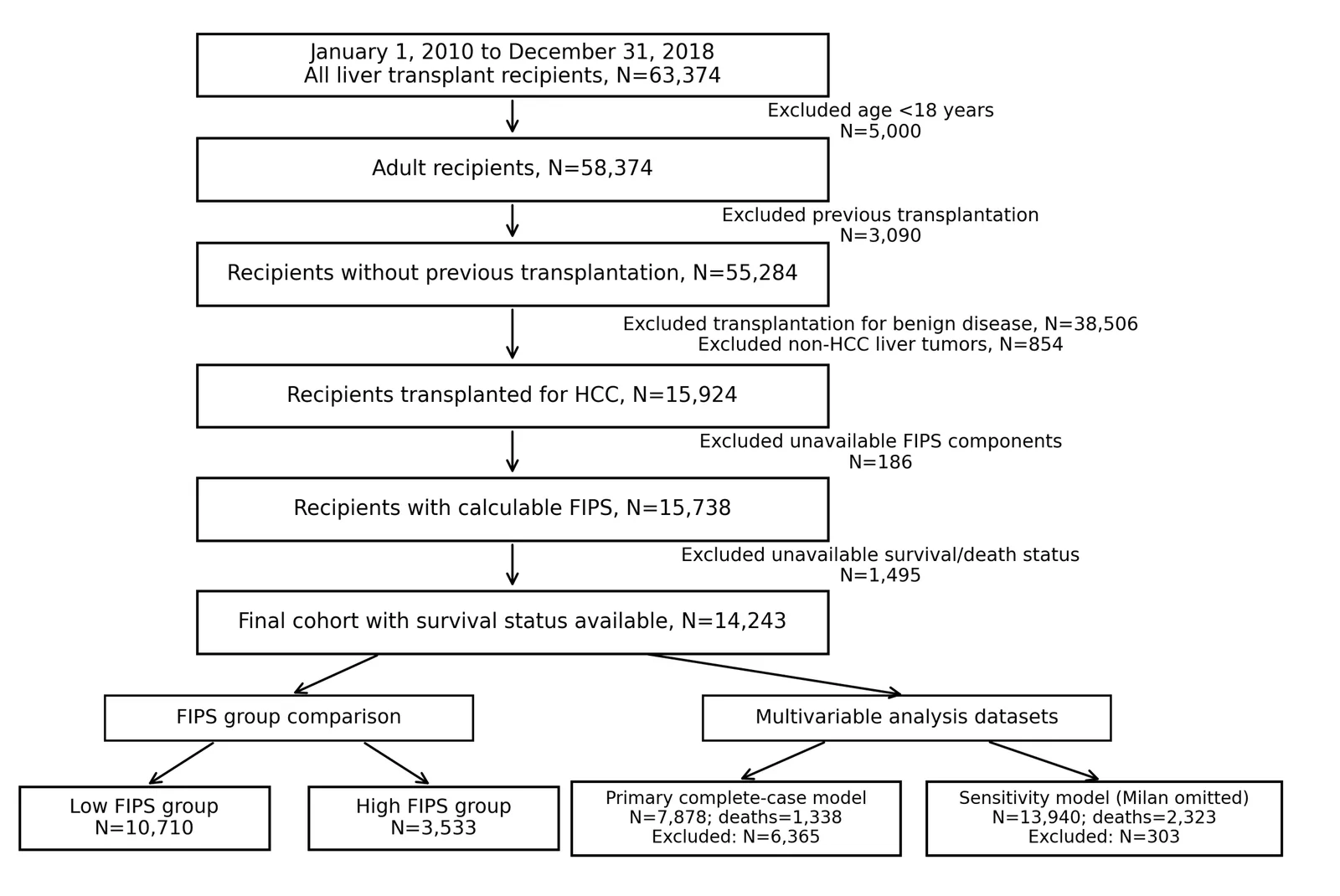
Patient selection flowchart for liver transplantation in hepatocellular carcinoma, including exclusions for unavailable FIPS components and unavailable survival or death status information FIPS: Freiburg Index of Post-TIPS Survival, HCC: hepatocellular carcinoma

The FIPS score was calculated as follows: FIPS = 1.43 × lg(total bilirubin) - 1.71 × (1/creatinine) + 0.02 × age - 0.02 × albumin + 0.81, with bilirubin and creatinine expressed in mg/dL, albumin in g/L, and age in years. To maintain comparability with previous FIPS studies, recipients were categorized using the previously reported cutoff of 0.92 (low FIPS: ≤0.92; high FIPS: >0.92) [9]. Because this cutoff was originally derived in a TIPS population, the categorical analysis was considered exploratory in the HCC transplant setting. The low- and high-FIPS groups included 10,710 and 3,533 recipients, respectively. FIPS was calculated from pre-transplant laboratory parameters and was evaluated as a pre-transplant prognostic marker.

Baseline recipient, donor, and tumor characteristics were compared between the low- and high-FIPS groups. The primary outcome was overall survival within three years after liver transplantation. FIPS was analyzed as a continuous variable in the primary multivariable model. Covariates were prespecified on the basis of clinical relevance and established associations with post-transplant outcomes and included recipient sex and race, donation after circulatory death, cold ischemia time, alpha-fetoprotein (AFP) ≥ 400 ng/mL, Milan criteria, hepatitis B virus (HBV) infection, hepatitis C virus (HCV) infection, non-alcoholic steatohepatitis (NASH), alcohol-associated liver disease, and transplant year. Age, bilirubin, creatinine, albumin, and MELD were not simultaneously included in the primary FIPS model because they are components of, or substantially overlap with, FIPS. A parallel model replacing FIPS with MELD was fitted using the same covariates and analytic sample.

Statistical analysis

Continuous variables were compared using the Kruskal-Wallis test and are reported as mean ± standard deviation or median (interquartile range), as appropriate. Categorical variables were compared using the chi-square test and are reported as counts and within-group percentages. Overall survival was estimated using the Kaplan-Meier method and compared with the log-rank test. Multivariable Cox regression was used to evaluate the association between FIPS and overall survival. The proportional hazards assumption was assessed using Schoenfeld residuals. Because FIPS violated this assumption, its effect was modeled using interval-specific coefficients for 0-1, 1-2, and 2-3 years. Collinearity was assessed using variance inflation factors. Complete-case analysis was performed without multiple imputation. The primary multivariable model included 7,878 recipients and 1,338 deaths. Because Milan criteria information was substantially incomplete, a sensitivity model excluding this variable was fitted in 13,940 recipients with 2,323 deaths.

Among the 14,243 recipients in the final cohort, 6,365 were excluded from the primary multivariable analysis because at least one adjustment covariate was missing, leaving 7,878 complete cases. Missing Milan criteria information accounted for most of this reduction and was unavailable for 6,255 (43.9%) recipients. The sensitivity analysis excluding Milan criteria therefore provided an assessment in a substantially larger analytic sample.

Discrimination of parallel FIPS- and MELD-based models was evaluated using Harrell's C-index and inverse-probability-of-censoring-weighted time-dependent area under the curves (AUCs) at one, two, and three years. Calibration plots and decision-curve analysis were used to assess agreement between predicted and observed mortality risks and potential clinical net benefit, respectively. Five-fold cross-validation was used for internal validation. Post-transplant HCC recurrence was analyzed using cumulative incidence functions and Fine-Gray competing-risk regression, with death without recurrence treated as a competing event; effect estimates are reported as subdistribution hazard ratios (sHRs). Restricted cubic spline analysis was used to visualize the association between continuous FIPS and all-cause mortality within each follow-up interval. All tests were two-sided, and P<0.05 was considered statistically significant. Analyses were performed using R version 4.2.1 (R Foundation for Statistical Computing, Vienna, Austria).

## Results

Baseline characteristics

The mean FIPS score in the overall cohort was 0.06. Compared with the low-FIPS group, recipients in the high-FIPS group were slightly older and had higher body weight, BMI, cold ischemia time, and substantially higher MELD scores. The distribution of recipient race and underlying liver disease also differed between groups. Tumor number and largest tumor diameter differed statistically between groups, whereas AFP level, total tumor diameter, and Milan criteria status did not. Donor characteristics were largely comparable, apart from the distribution of donor ABO blood group. Detailed baseline characteristics are presented in Table 1.

**Table 1:** Baseline characteristics of liver transplant recipients according to pre-transplant FIPS group Data are presented as mean ± standard deviation, median (interquartile range), or number (%), as appropriate. Percentages for categorical variables were calculated using the total number of recipients within each FIPS group as the denominator; missing or unknown observations are shown separately where applicable. P values for donation after circulatory death, AFP, and Milan criteria were calculated using non-missing categories. AFP: alpha-fetoprotein, BMI: body mass index, DCD: donation after circulatory death, FIPS: Freiburg Index of Post-TIPS Survival, HBV: hepatitis B virus, HCV: hepatitis C virus, MELD: Model for End-Stage Liver Disease, NASH: non-alcoholic steatohepatitis

Characteristic	High FIPS (n=3,533)	Low FIPS (n=10,710)	P value
Donor variables			
Donor race			0.784
Black	611 (17.3%)	1915 (17.9%)	
White	2785 (78.8%)	8381 (78.3%)	
Asian	96 (2.7%)	308 (2.9%)	
Native	15 (0.4%)	42 (0.4%)	
Other	26 (0.7%)	64 (0.6%)	
Donor ABO			0.010
O	1605 (45.4%)	4895 (45.7%)	
A	1398 (39.6%)	3994 (37.3%)	
B	444 (12.6%)	1485 (13.9%)	
AB	86 (2.4%)	336 (3.1%)	
Donor sex			0.714
Female	1419 (40.2%)	4339 (40.5%)	
Male	2114 (59.8%)	6371 (59.5%)	
Donor height (cm)	171.50 ± 10.97	171.24 ± 10.77	0.210
Donor weight (kg)	82.73 ± 21.08	82.17 ± 20.74	0.165
Donation after circulatory death			0.068
No	3260 (92.3%)	9773 (91.3%)	
Yes	225 (6.4%)	778 (7.3%)	
Unknown	48 (1.4%)	159 (1.5%)	
Recipient variables			
Recipient age (years)	60.61 ± 6.80	60.24 ± 6.92	0.006
Recipient sex			0.067
Male	2756 (78.0%)	8194 (76.5%)	
Female	777 (22.0%)	2516 (23.5%)	
Recipient race			<0.001
White	3008 (85.1%)	8621 (80.5%)	
Black	302 (8.5%)	1072 (10.0%)	
Asian	167 (4.7%)	875 (8.2%)	
Other	29 (0.8%)	70 (0.7%)	
Native	27 (0.8%)	72 (0.7%)	
Recipient ABO			0.016
O	1576 (44.6%)	4851 (45.3%)	
A	1366 (38.7%)	3927 (36.7%)	
B	474 (13.4%)	1465 (13.7%)	
AB	117 (3.3%)	467 (4.4%)	
Recipient height (cm)	172.88 ± 9.90	172.18 ± 10.07	<0.001
Recipient weight (kg)	87.12 ± 18.99	85.40 ± 18.25	<0.001
Cold ischemia time (hours)	6.32 ± 2.60	6.14 ± 2.58	<0.001
Warm ischemia time (minutes)	38.0 (30.0, 48.0)	38.0 (30.0, 47.0)	0.863
Recipient BMI (kg/m²)	29.16 ± 8.27	28.76 ± 5.99	0.008
Laboratory MELD score	21.33 ± 7.59	10.61 ± 3.59	<0.001
FIPS score	2.12 ± 1.19	-0.62 ± 0.95	<0.001
HBV surface antigen			<0.001
Negative	3379 (95.6%)	9947 (92.9%)	
Positive	154 (4.4%)	763 (7.1%)	
HCV serology			<0.001
Positive	1899 (53.8%)	6564 (61.3%)	
Negative	1634 (46.2%)	4146 (38.7%)	
NASH			<0.001
Negative	3251 (92.0%)	10235 (95.6%)	
Positive	282 (8.0%)	475 (4.4%)	
Alcohol-associated liver disease			<0.001
Negative	3224 (91.3%)	10066 (94.0%)	
Positive	309 (8.7%)	644 (6.0%)	
Tumor variables			
AFP (ng/mL)			0.563
<400	3400 (96.2%)	10347 (96.6%)	
≥400	104 (2.9%)	296 (2.8%)	
Missing	29 (0.8%)	67 (0.6%)	
Tumor number	1.44 ± 0.80	1.33 ± 0.68	<0.001
Total tumor diameter (cm)	3.49 ± 1.88	3.48 ± 1.98	0.863
Largest tumor diameter (cm)	2.79 ± 1.21	2.94 ± 1.50	<0.001
Milan criteria			0.287
Within Milan	1580 (44.7%)	5809 (54.2%)	
Beyond Milan	117 (3.3%)	482 (4.5%)	
Unknown	1836 (52.0%)	4419 (41.3%)	

Overall survival

Overall survival differed significantly between the FIPS groups. In the low-FIPS group, the one-, two-, and three-year OS rates were 93.1%, 88.3%, and 83.8%, respectively, compared with 89.5%, 84.7%, and 79.6% in the high-FIPS group (Figure 2A-2C). Cardiovascular events were the most frequently recorded cause of death within 30 days after transplantation (Table 2). In subgroup analyses according to underlying liver disease, survival differed significantly between FIPS groups among recipients with HCV infection, HBV infection, and alcohol-associated liver disease, whereas no significant difference was observed in the NASH subgroup (Figure 2D-2G).

**Figure 2:**
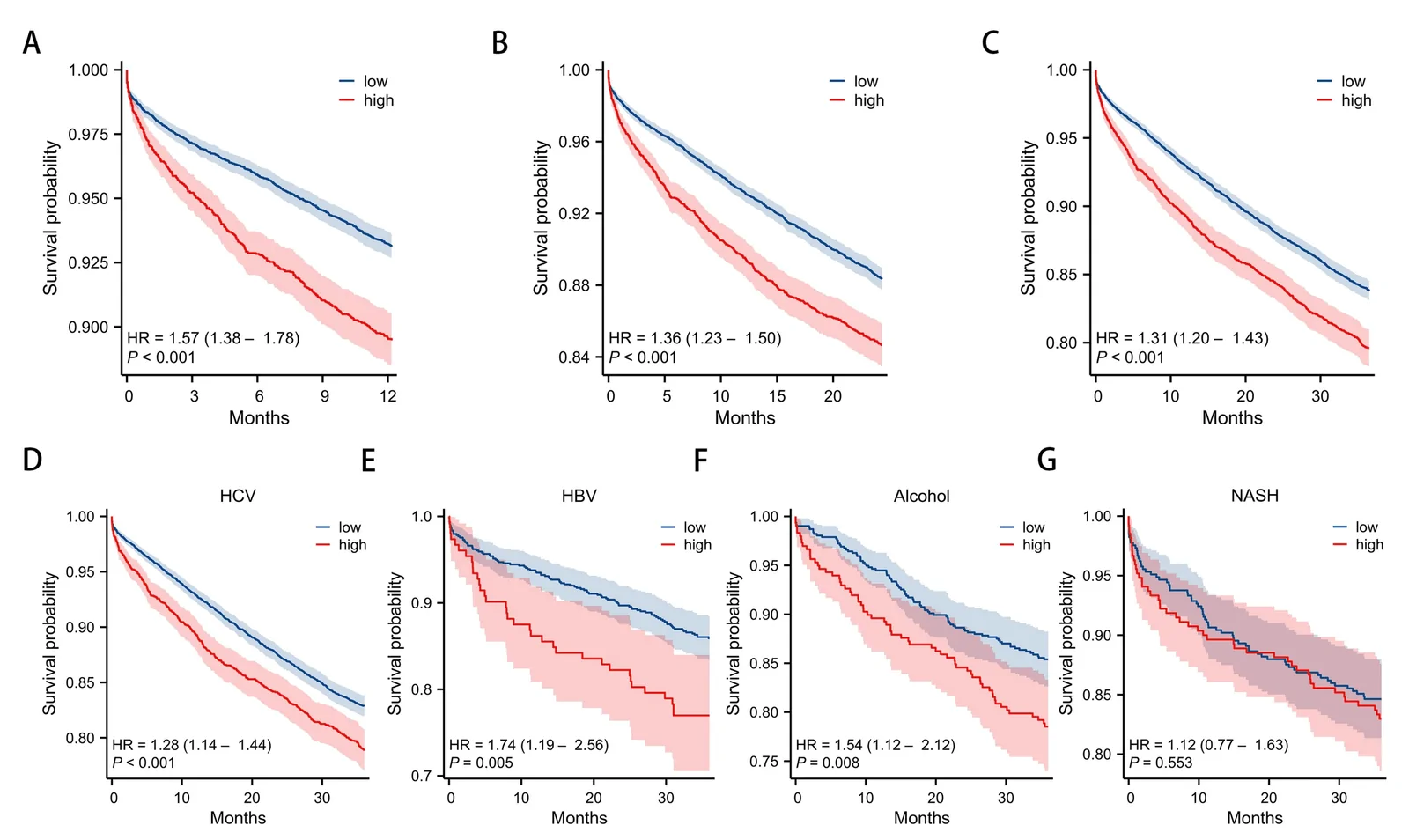
Overall survival rates by FIPS score and underlying liver diseases (A-C) OS comparisons between low- and high-FIPS groups at one, two, and three years; (D-F) OS among patients with HCV, HBV, and alcoholic liver disease; and (G) OS in NASH subgroup participants FIPS: Freiburg Index of Post-TIPS Survival, HBV: hepatitis B virus, HCV: hepatitis C virus, NASH: non-alcoholic steatohepatitis, OS: overall survival

**Table 2:** Primary causes of death within 30 days after transplantation HCC: hepatocellular carcinoma

Mortality within 30 days			Mortality within 3 years		
Characteristics	Low risk (n=183)	High risk (n=103)	Characteristics	Low risk (n=1671)	High risk (n=701)
Cardiovascular/cerebrovascular, number (%)	69 (37.7.1%)	48 (46.6%)	Cardiovascular/cerebrovascular, number (%)	189 (11.3%)	106 (15.1%)
Graft failure, number (%)	27 (14.8%)	12 (11.7%)	Graft failure, number (%)	133 (8.0%)	50 (7.1%)
Organ failure, number (%)	23 (12.6%)	12 (11.7%)	Organ failure, number (%)	218 (13.0%)	98 (14.0%)
Hemorrhage, number (%)	16 (8.7%)	8 (7.8%)	Hemorrhage, number (%)	40 (2.4%)	16 (2.3%)
Infection, number (%)	17 (9.3%)	13 (12.6%)	Infection, number (%)	158 (9.5%)	76 (10.8%)
HCC recurrence, number (%)	0 (0%)	2 (1.9%)	HCC recurrence, number (%)	519 (31.1%)	193 (27.5%)
Others, number (%)	31 (16.9%)	8 (7.8%)	Others, number (%)	411 (24.6%)	161 (23.0%)

Multivariable analysis of overall survival

The primary complete-case analysis included 7,878 recipients and 1,338 deaths. In the adjusted time-varying Cox model, each one-unit increase in FIPS was associated with a hazard ratio (HR) of 1.125 (95% confidence interval (CI): 1.069-1.183; P<0.001) during 0-1 year, 1.038 (95% CI: 0.973-1.107; P=0.254) during 1-2 years, and 1.112 (95% CI: 1.040-1.190; P=0.002) during 2-3 years. Black race versus White race (HR: 1.334; 95% CI: 1.135-1.568; P<0.001), Native race versus White race (HR: 1.685; 95% CI: 1.027-2.765; P=0.039), AFP ≥ 400 ng/mL (HR: 1.697; 95% CI: 1.285-2.240; P<0.001), and transplant year (HR per year: 0.956; 95% CI: 0.935-0.976; P<0.001) were also independently associated with overall survival (Table 3).

**Table 3:** Time-varying multivariable Cox regression analysis of three-year overall survival after liver transplantation The primary complete-case analysis included 7,878 recipients and 1,338 deaths. Because FIPS violated the proportional hazards assumption, its effect was modeled using interval-specific coefficients. The reported FIPS HRs represent the change in mortality hazard associated with each one-unit increase in FIPS. The model was adjusted for recipient sex and race, donation after circulatory death, cold ischemia time, AFP ≥ 400 ng/mL, Milan criteria, HBV, HCV, NASH, alcohol-associated liver disease, and transplant year. Age, bilirubin, creatinine, albumin, and MELD were not simultaneously included because they are components of or substantially overlap with FIPS. AFP: alpha-fetoprotein, CI: confidence interval, FIPS: Freiburg Index of Post-TIPS Survival, HBV: hepatitis B virus, HCV: hepatitis C virus, HR: hazard ratio, MELD: Model for End-Stage Liver Disease, NASH: non-alcoholic steatohepatitis

Variable	Comparison or interval	Adjusted HR (95% CI)	P value
FIPS (per one-unit increase)	0-1 year	1.125 (1.069-1.183)	<0.001
FIPS (per one-unit increase)	1-2 years	1.038 (0.973-1.107)	0.254
FIPS (per one-unit increase)	2-3 years	1.112 (1.040-1.190)	0.002
Recipient sex	Female versus male	0.970 (0.850-1.108)	0.656
Recipient race	Black versus White	1.334 (1.135-1.568)	<0.001
	Asian versus White	0.820 (0.640-1.051)	0.117
	Native versus White	1.685 (1.027-2.765)	0.039
	Other versus White	0.735 (0.381-1.419)	0.359
Donation after circulatory death	Yes versus no	1.078 (0.880-1.320)	0.469
Cold ischemia time		1.001 (0.980-1.022)	0.927
AFP	≥400 versus <400 ng/mL	1.697 (1.285-2.240)	<0.001
Milan criteria	Beyond versus within Milan	1.203 (0.993-1.458)	0.059
HBV	Positive versus negative	1.122 (0.880-1.429)	0.353
HCV	Positive versus negative	1.096 (0.969-1.239)	0.145
NASH	Positive versus negative	0.991 (0.747-1.314)	0.949
Alcohol-associated liver disease	Present versus absent	0.981 (0.789-1.221)	0.866
Transplant year		0.956 (0.935-0.976)	<0.001

All variance inflation factors (VIFs) were below 1.34 (maximum VIF: 1.337), indicating no substantial multicollinearity among the variables retained in the revised primary model (Table 4).

**Table 4:** VIFs for variables included in the revised primary FIPS model The maximum VIF was 1.337. All VIF values were below 1.34, indicating no substantial multicollinearity among variables retained in the revised model. AFP: alpha-fetoprotein, DCD: donation after circulatory death, FIPS: Freiburg Index of Post-TIPS Survival, HBV: hepatitis B virus, HCV: hepatitis C virus, NASH: non-alcoholic steatohepatitis, VIF: variance inflation factor

Variable or contrast	VIF
FIPS	1.031
Female versus male	1.029
Recipient race: Black versus White	1.042
Recipient race: Asian versus White	1.311
Recipient race: Native versus White	1.006
Recipient race: other versus White	1.005
DCD: yes versus no	1.007
Cold ischemia time, per hour	1.014
AFP ≥400 versus <400	1.013
Beyond versus within Milan	1.005
HBV positive versus negative	1.337
HCV positive versus negative	1.208
NASH positive versus negative	1.115
Alcohol-related versus not	1.031
Transplant year, per year	1.036

In the sensitivity analysis excluding Milan criteria, 13,940 recipients and 2,323 deaths were included. The adjusted HRs per one-unit increase in FIPS were 1.134 (95% CI: 1.094-1.175; P<0.001) during 0-1 year, 1.052 (95% CI: 1.003-1.104; P=0.038) during 1-2 years, and 1.058 (95% CI: 1.005-1.114; P=0.031) during 2-3 years. Thus, the sensitivity analysis yielded directionally consistent associations and supported the robustness of the primary findings (Table 5).

**Table 5:** Sensitivity analysis of the interval-specific association between pre-transplant FIPS and three-year overall survival after excluding Milan criteria from the adjustment set FIPS was modeled using interval-specific coefficients in both models. The primary model included Milan criteria, whereas the sensitivity model omitted Milan criteria because of substantial missingness. Both models were adjusted for recipient sex and race, donation after circulatory death, cold ischemia time, AFP ≥ 400 ng/mL, HBV infection, HCV infection, NASH, alcohol-associated liver disease, and transplant year. The primary model additionally included Milan criteria. The sensitivity estimates were directionally consistent with the primary analysis, although the magnitude and statistical significance of the associations differed during the later follow-up intervals. AFP: alpha-fetoprotein, CI: confidence interval, FIPS: Freiburg Index of Post-TIPS Survival, HBV: hepatitis B virus, HCV: hepatitis C virus, HR: hazard ratio, NASH: non-alcoholic steatohepatitis

Analysis	Interval	Number	Deaths	Milan criteria	Adjusted HR per one-unit increase in FIPS (95% CI)	P value
Primary model	0-1 year	7,878	1,338	Included	1.125 (1.069-1.183)	<0.001
Primary model	1-2 years	7,878	1,338	Included	1.038 (0.973-1.107)	0.254
Primary model	2-3 years	7,878	1,338	Included	1.112 (1.040-1.190)	0.002
Sensitivity model	0-1 year	13,940	2,323	Excluded	1.134 (1.094-1.175)	<0.001
Sensitivity model	1-2 years	13,940	2,323	Excluded	1.052 (1.003-1.104)	0.038
Sensitivity model	2-3 years	13,940	2,323	Excluded	1.058 (1.005-1.114)	0.031

Time‑dependent effect of FIPS on OS

Schoenfeld residual analysis showed evidence of non-proportional hazards for FIPS (P=0.0017), and the global test was also significant (P=0.0066). No statistically significant evidence of proportional hazards violation was detected for the other individual covariates. Accordingly, the association between FIPS and overall survival was characterized using interval-specific estimates for 0-1, 1-2, and 2-3 years rather than a single constant HR (Table 3 and Table 6). The corresponding interval-specific effects of MELD are presented in Table 7.

**Table 6:** Interval-specific effects of pre-transplant FIPS on overall survival estimated using a time-varying Cox regression model FIPS: Freiburg Index of Post-TIPS Survival

Term	HR	Lower 95	Upper 95	P value
FIPS: 0-1 year	1.125	1.069	1.183	<0.001
FIPS: 1-2 years	1.038	0.973	1.107	0.254
FIPS: 2-3 years	1.112	1.040	1.190	0.002

**Table 7:** Interval-specific effects of pre-transplant MELD on overall survival estimated using a time-varying Cox regression model MELD: Model for End-Stage Liver Disease

Term	HR	Lower 95	Upper 95	P value
MELD: 0-1 year	1.030	1.019	1.041	<0.001
MELD: 1-2 years	1.004	0.989	1.019	0.623
MELD: 2-3 years	1.020	1.005	1.036	0.010

Post‑transplant tumor recurrence

A total of 657 recipients experienced post-transplant HCC recurrence, including 321 recurrences within three years. Recurrence was evaluated using a Fine-Gray competing-risk model, with death without recurrence treated as the competing event. The point estimate indicated a numerically higher recurrence risk in the high-FIPS group than in the low-FIPS group, although the association did not reach statistical significance (sHR: 1.22; 95% CI: 0.97-1.54; P=0.095; Figure 3 and Table 8).

**Figure 3:**
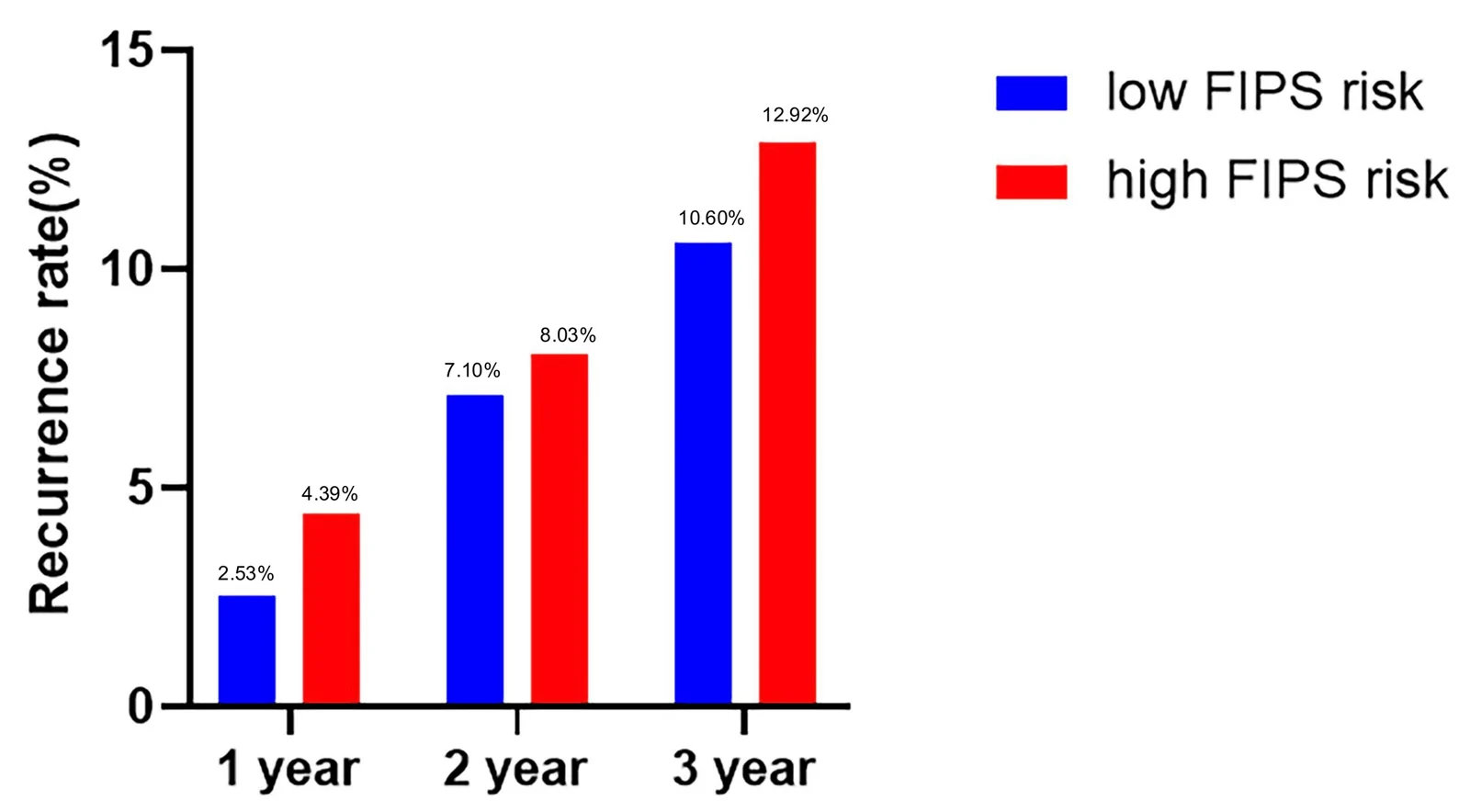
Cumulative incidence of post-transplant HCC recurrence according to FIPS group, with death without recurrence treated as a competing event FIPS: Freiburg Index of Post-TIPS Survival, HCC: hepatocellular carcinoma

**Table 8:** Fine-Gray competing-risk regression analysis of post-transplant HCC recurrence according to FIPS group Death without HCC recurrence was treated as a competing event. The low-FIPS group served as the reference. CI: confidence interval, FIPS: Freiburg Index of Post-TIPS Survival, HCC: hepatocellular carcinoma, sHR: subdistribution hazard ratio

Variable	Comparison	sHR	95% CI	P value
FIPS group	High versus low	1.22	0.97-1.54	0.095

Restricted cubic spline regression analysis

Restricted cubic spline analysis was used to visualize the association between continuous pre-transplant FIPS and all-cause mortality across the prespecified follow-up intervals (Figure 4). Separate curves were estimated for 0-1, 1-2, and 2-3 years because the effect of FIPS varied over time. The interval-specific estimates were consistent with the time-varying Cox model: higher FIPS was significantly associated with mortality during 0-1 year (HR: 1.125; 95% CI: 1.069-1.183; P<0.001) and 2-3 years (HR: 1.112; 95% CI: 1.040-1.190; P=0.002), whereas the association was attenuated during 1-2 years (HR: 1.038; 95% CI: 0.973-1.107; P=0.254). Figure 4 evaluates all-cause mortality and was not used to infer HCC recurrence risk.

**Figure 4:**
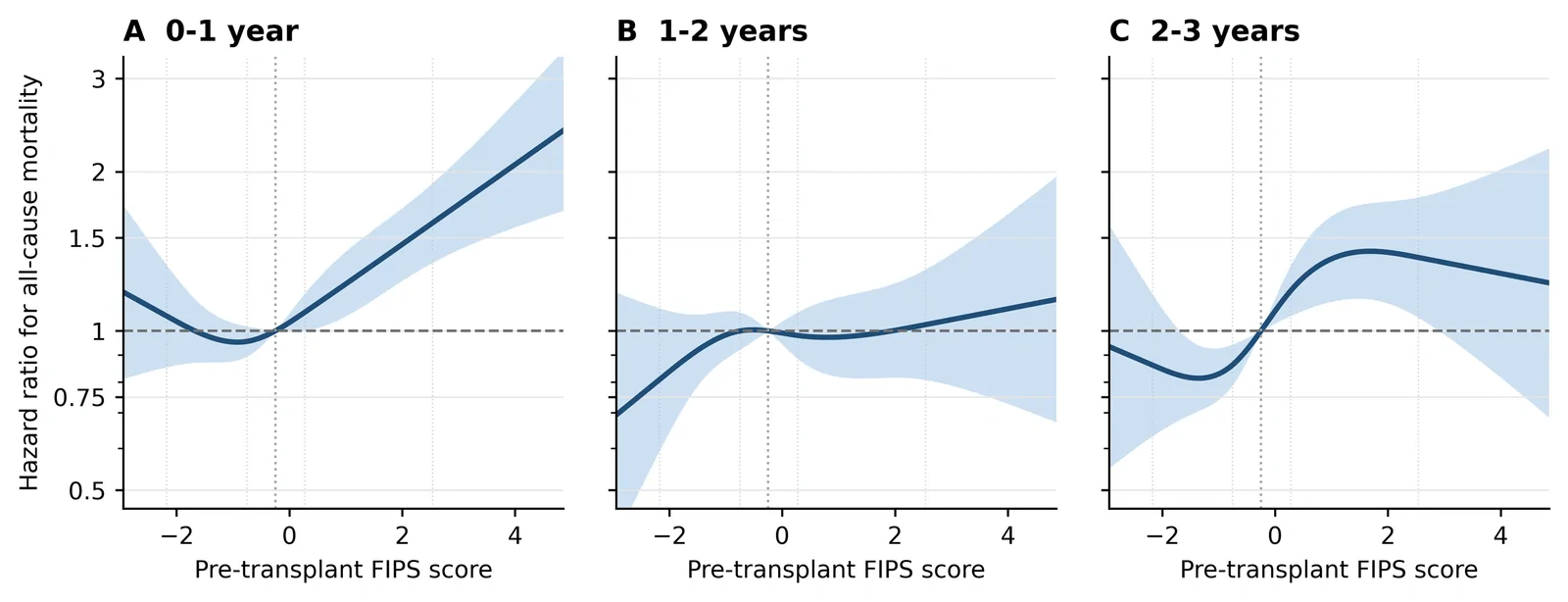
Interval-specific restricted cubic spline analysis of the association between continuous pre-transplant FIPS and all-cause mortality after liver transplantation for hepatocellular carcinoma Separate curves were estimated for 0-1 year (A), 1-2 years (B), and 2-3 years (C). Solid lines represent adjusted hazard ratios, shaded areas represent 95% confidence intervals, and horizontal dashed lines indicate HR=1. Figure 4 evaluates overall survival rather than HCC recurrence. FIPS: Freiburg Index of Post-TIPS Survival, HCC: hepatocellular carcinoma, HR: hazard ratio

Comparative prognostic performance of FIPS and MELD

In the same complete-case cohort of 7,878 recipients, the apparent Harrell's C-indices were 0.573 for the FIPS-based model and 0.571 for the MELD-based model; the corresponding five-fold cross-validated C-indices were 0.564 and 0.563, respectively. Cross-validated time-dependent AUCs for the FIPS model were 0.565, 0.573, and 0.563 at one, two, and three years, compared with 0.569, 0.573, and 0.560 for the MELD model. Calibration plots showed similar agreement between predicted and observed mortality risks, and the decision curves were largely overlapping. Taken together, the FIPS- and MELD-based models demonstrated closely comparable prognostic performance across multiple evaluation metrics (Figures 5-7 and Table 9).

**Figure 5:**
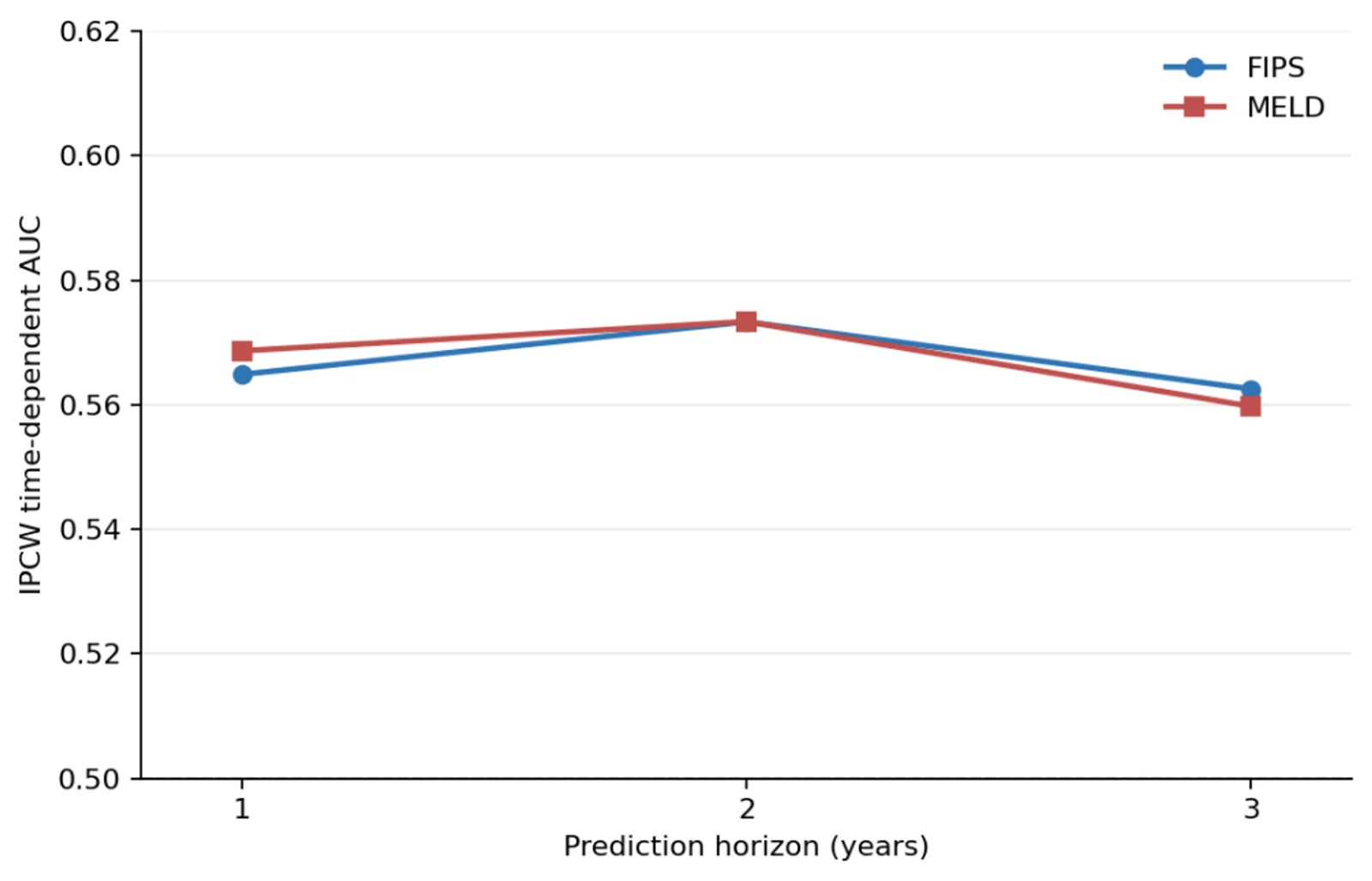
Five-fold cross-validated IPCW time-dependent AUCs of the FIPS- and MELD-based models at one, two, and three years after liver transplantation AUC: area under the curve, FIPS: Freiburg Index of Post-TIPS Survival, IPCW: inverse probability of censoring weighting, MELD: Model for End-Stage Liver Disease

**Figure 6:**
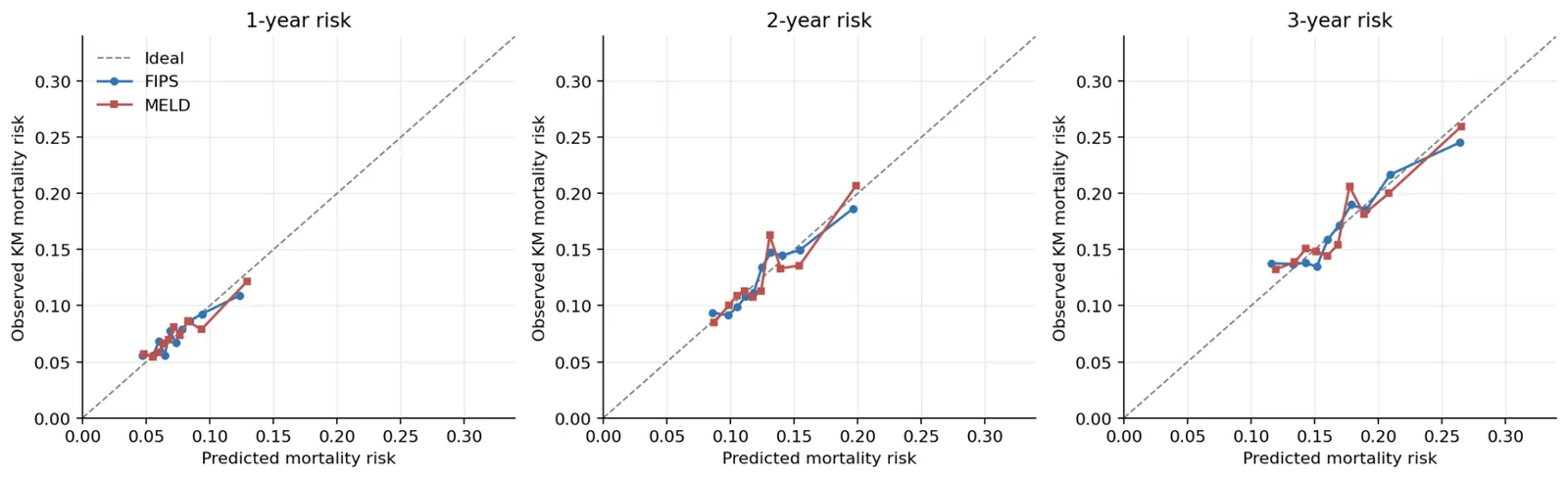
Five-fold cross-validated calibration plots comparing predicted and Kaplan-Meier-estimated mortality risks at one, two, and three years after liver transplantation The dashed diagonal line represents perfect calibration.

**Figure 7:**
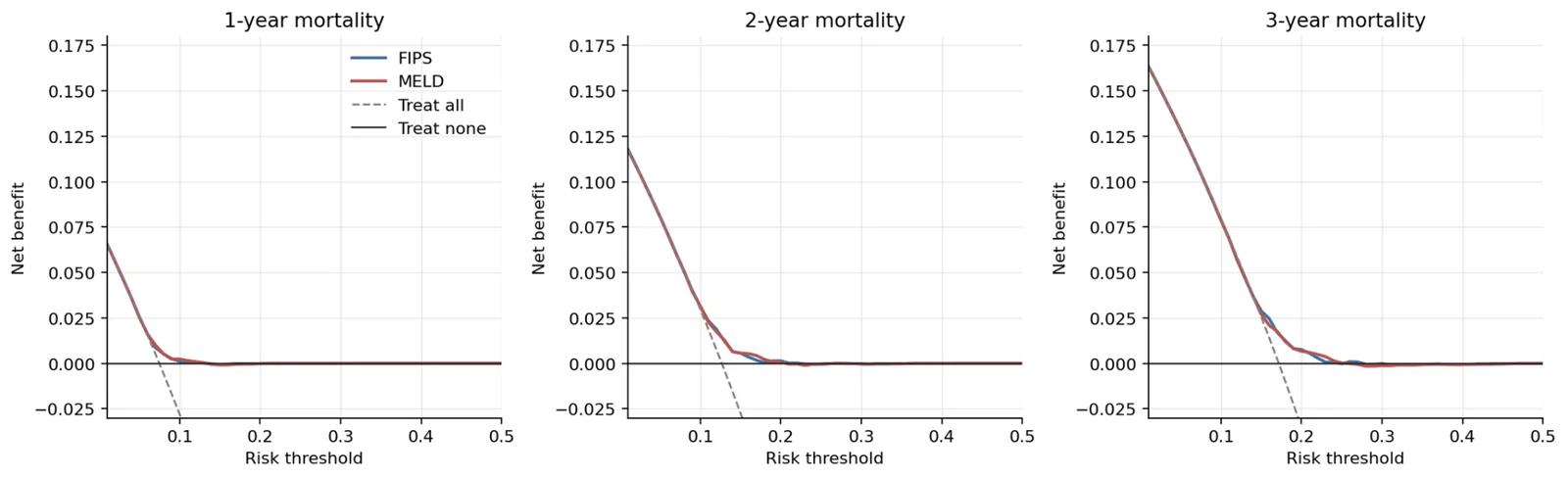
Five-fold cross-validated decision-curve analysis comparing the clinical net benefits of the FIPS- and MELD-based models across different risk thresholds The dashed and solid reference lines represent the treat-all and treat-none strategies, respectively. FIPS: Freiburg Index of Post-TIPS Survival, MELD: Model for End-Stage Liver Disease

**Table 9:** Comparative prognostic performance of the FIPS- and MELD-based models for three-year mortality after liver transplantation Time-dependent AUCs were estimated using inverse probability of censoring weighting. Internal validation was performed using five-fold cross-validation. Both models were fitted in the same complete-case sample and used identical adjustment covariates, with either FIPS or MELD as the principal predictor. AUC: area under the curve, CV: cross-validation, FIPS: Freiburg Index of Post-TIPS Survival, MELD: Model for End-Stage Liver Disease

Model	Apparent Harrell's C-index	5-fold CV Harrell's C-index	1-year CV AUC	2-year CV AUC	3-year CV AUC
FIPS	0.573	0.564	0.565	0.573	0.563
MELD	0.571	0.563	0.569	0.573	0.560

## Discussion

In this large registry-based cohort of patients undergoing LT for HCC, pre-transplant FIPS was associated with early post-transplant overall survival, and the magnitude of this association varied over time. Higher FIPS was independently associated with increased mortality during the first post-transplant year and again during years 2-3 in the primary analysis. Importantly, the sensitivity analysis excluding Milan criteria included a substantially larger sample and demonstrated significant associations across all three follow-up intervals. FIPS- and MELD-based models showed closely comparable prognostic performance, while the recurrence analysis suggested a higher point estimate in the high-FIPS group without reaching statistical significance after accounting for the competing risk of death.

Risk assessment after LT for HCC has traditionally focused on tumor-related characteristics, including tumor size, number, differentiation, vascular invasion, and biomarkers such as AFP [12]. At the same time, recipient physiologic reserve and the severity of underlying liver disease remain important determinants of perioperative and early post-transplant outcomes. MELD and related models are well established for the assessment of liver disease severity and organ allocation priority [8,13], whereas FIPS was developed to integrate age and markers of hepatic and renal function for prognostic assessment after TIPS [9-11]. Our findings extend the potential application of FIPS to the pre-transplant setting in HCC and show that its survival association is not constant over time. Rather than replacing MELD, FIPS may offer a complementary summary of recipient physiologic status that can be interpreted alongside tumor-related and transplant-specific factors.

The components of FIPS provide a biologically plausible basis for its association with post-transplant survival. Serum albumin reflects hepatic synthetic function and is also influenced by nutritional and systemic inflammatory status [14,15]. Bilirubin is a clinically established marker of hepatic excretory function, although it may also be affected by factors beyond intrinsic liver function [14]. Age captures aspects of physiologic reserve and is closely related to frailty and comorbidity burden among transplant candidates and recipients [16-19]. Creatinine reflects renal function, which is an important determinant of outcomes before and after LT [20-22]. By integrating these four readily available variables, FIPS summarizes several dimensions of recipient reserve that may contribute to early post-transplant survival. Several established models, including RETREAT and Metroticket 2.0, have been developed to assess tumor-related risk in HCC transplant candidates using tumor burden and biological markers, while molecular imaging offers additional insights into tumor biology beyond conventional anatomical imaging [23-25]. The comparable performance of FIPS and MELD in the present analysis suggests that FIPS may be most useful as an adjunctive marker rather than as an isolated decision tool.

We also explored the prognostic pattern of FIPS across major etiologic subgroups. Survival differed between low- and high-FIPS recipients with HCV infection, HBV infection, and alcohol-associated liver disease, whereas no significant difference was observed in the NASH subgroup. Previous studies have shown that the impact of clinical risk factors after LT may vary according to the underlying liver disease [26], and FIPS has demonstrated prognostic value in cohorts dominated by viral cirrhosis [27]. These subgroup findings suggest that the relationship between FIPS and post-transplant outcomes may be influenced by disease etiology and merit further evaluation in independent cohorts.

The recurrence analysis provides a complementary perspective on the prognostic role of FIPS. Although the high-FIPS group had a higher point estimate for post-transplant HCC recurrence, the association did not reach statistical significance after death without recurrence was treated as a competing event. This pattern suggests that the prognostic information captured by FIPS may relate more strongly to recipient survival and physiologic reserve than to tumor recurrence itself. Studies incorporating more detailed tumor biology, explant pathology, and post-transplant treatment information will be important for clarifying this relationship.

Several considerations should be acknowledged when interpreting these findings. First, the SRTR database did not provide complete information on some tumor-related variables, including microvascular invasion and Edmondson-Steiner grade, and detailed data on contemporary targeted or immune-based therapies were unavailable. Second, the 0.92 FIPS cutoff was derived in a TIPS population and has not been specifically optimized for HCC transplant recipients; therefore, the categorical analysis should be considered exploratory. Third, missing Milan criteria information reduced the primary complete-case sample, although the larger sensitivity analysis excluding this variable produced directionally consistent and statistically significant FIPS estimates across all follow-up intervals. Finally, the study used internal rather than external validation, and the time-varying association of FIPS indicates that its prognostic effect should be interpreted according to the post-transplant interval.

Overall, this large national registry analysis shows that pre-transplant FIPS captures clinically relevant variation in early survival after LT for HCC and performs comparably to MELD across multiple prognostic metrics. The consistency of the sensitivity analysis supports the robustness of the survival association, while the time-dependent findings provide additional information on how the prognostic effect evolves during the first three years after transplantation. These results support further evaluation of FIPS as a complementary component of pre-transplant risk assessment in HCC.

## Conclusions

Pre-transplant FIPS was associated with early overall survival after liver transplantation for HCC, with a prognostic effect that varied across follow-up intervals. FIPS-based risk prediction showed performance comparable to that of MELD, and the association with survival was supported by a larger sensitivity analysis. These findings support further evaluation of FIPS as a complementary pre-transplant risk stratification marker in patients with HCC undergoing liver transplantation. The relationship between FIPS and post-transplant HCC recurrence requires additional study.
